# c-Abl Is an Upstream Regulator of Acid Sphingomyelinase in Apoptosis Induced by Inhibition of Integrins αvβ3 and αvβ5

**DOI:** 10.1371/journal.pone.0042291

**Published:** 2012-08-03

**Authors:** Xiuhai Ren, Jingying Xu, Jason P. Cooper, Min H. Kang, Anat Erdreich-Epstein

**Affiliations:** 1 Division of Hematology-Oncology, Department of Pediatrics, Keck School of Medicine, University of Southern California and the Saban Research Institute of Children’s Hospital Los Angeles, Los Angeles, California, United States of America; 2 School of Medicine, Texas Tech University Health Sciences Center, Lubbock, Texas, United States of America; 3 Department of Pathology, Keck School of Medicine, University of Southern California and the Saban Research Institute of Children’s Hospital Los Angeles, Los Angeles, California, United States of America; MUSC SC College of Pharmacy, United States of America

## Abstract

Inhibition of integrins αvβ3/αvβ5 by the cyclic function-blocking peptide, RGDfV (Arg-Gly-Asp-Phe-Val) can induce apoptosis in both normal cells and tumor cells. We show that RGDfV induced apoptosis in ECV-304 carcinoma cells, increased activity and mRNA expression of acid sphingomyelinase (ASM), and increased ceramides C_16_, C_18∶0_, C_24∶0_ and C_24∶1_ while decreasing the corresponding sphingomyelins. siRNA to ASM decreased RGDfV-induced apoptosis as measured by TUNEL, PARP cleavage, mitochondrial depolarization, and caspase-3 and caspase-8 activities, as well as by annexinV in a 3D collagen model. These findings indicate a causal role for ASM in RGDfV-induced apoptosis in ECV-304. We have shown that c-Abl, a non-receptor tyrosine kinase, also mediates RGDfV-induced apoptosis. However, c-Abl, has not been previously linked to ASM in any system. Here we show that STI-571 (imatinib, inhibitor of c-Abl) inhibited RGDfV-induced ASM activity. Furthermore, STI-571 and c-Abl-siRNA both inhibited RGDfV-induced increase in ASM mRNA, but ASM-siRNA did not affect c-Abl phosphorylation or expression, supporting that c-Abl regulates the RGDfV-induced increase in ASM expression. These studies implicate ASM as a mediator of apoptosis induced by inhibition of integrins αvβ3/αvβ5, and for the first time place c-Abl as an upstream regulator of ASM expression and activity.

## Introduction

Integrins, heterodimeric cell-surface receptors, are central regulators of cell functions such as proliferation, differentiation, growth factor secretion and protection from apoptosis [1], [2], [3]. Integrins αvβ3 and αvβ5 are expressed on a variety of cell types including cancer cells and endothelial cells [3], [4], [5], [6]. Inhibition of integrins αvβ3 and αvβ5 can induce cell death and affect tumor growth [7], [8], [9], [10]. Integrins αvβ3 and αvβ5 bind to arginine-glycine-aspartic acid (RGD)-containing matrix proteins such as vitronectin. Integrin αvβ3/αvβ5 signaling can be blocked by soluble function-blocking RGD peptides such as the cyclic RGDfV (Arg-Gly-Asp-Phe-Val) peptide, resulting in apoptosis [8], [9], [11]. *In vivo* RGDfV inhibits growth of cell line-derived tumors such as glioblastoma, medulloblastoma, and breast cancer in mice [3], [12]. Moreover, the clinical version of RGDfV, Cilengitide, is in clinical trials [12], [13], [14], [15], underscoring the need to fully understand the molecular mechanism(s) that are affected by RGDfV.

Ceramide, an intracellular sphingolipid second messenger, can be increased by pro-apoptotic stimuli such as UV, ionizing irradiation and lipopolysaccharide [10], [16], [17], [18], [19], [20], [21], and is thought to have pro-apoptotic function. Two central pathways for generation of ceramide in apoptosis are *de novo* synthesis starting with condensation of palmitoyl-CoA to serine, catalyzed by serine palmitoyltransferase, and hydrolysis of sphingomyelin by sphingomyelinases [17], [18], [22], [23]. Acid sphingomyelinase (ASM) can mediate apoptosis induced by stimuli such as irradiation, lipopolysaccharide (LPS), and others [24], [25], [26]. Using thin layer chromatography and pharmacological inhibitors, we have shown that inhibition of integrins αvβ3/αvβ5 by RGDfV increases incorporation of [3H]palmitic acid into ceramide species and is associated with apoptosis [11], [27]. In that setting, the nonspecific ASM inhibitors desipramine, imipramine (tricyclic antidepressants) and SR33557 (a calcium channel blocker) decreased apoptosis induced by RGDfV, suggesting that ASM may be the mediator of the increase in ceramide, and that this sphingolipid pathway may be required for RGDfV-induced apoptosis [11]. However, these inhibitors have functions other than inhibition of ASM, and therefore, a role for ASM in RGDfV-induced apoptosis remained inconclusive.

c-Abl is a non-receptor tyrosine kinase mostly known for its proliferative and oncogenic potential. c-Abl is clinically important as the constitutively-active kinase in the oncogenic fusion protein BCR-ABL1 in chronic myelogenous leukemia and in some acute lymphoblastic leukemias [28], [29]. Interestingly, c-Abl can also mediate apoptosis induced by stimuli such as DNA damage-inducing agents and disruption of cell shape, and we recently showed that it was required for apoptosis induced by inhibition of integrins αvβ3/αvβ5 by RGDfV [30], [31], [32], [33]. However, it is completely unknown whether the molecular mechanism of c-Abl and ASM in RGDfV-induced apoptosis are interconnected.

In the work presented here we addressed two questions: 1) does ASM mediate RGDfV-induced apoptosis, and 2) do ASM and c-Abl function in separate pathways or in the same apoptotic signaling pathway initiated by RGDfV, and if the latter, what is their molecular ordering. Our data now show that inhibition of integrins αvβ3/αvβ5 by RGDfV, which induced ECV-304 apoptosis, increased ASM activity and mRNA expression, and that this ASM increase was required for apoptosis. Further, while c-Abl inhibition and knockdown blocked the RGDfV-induced increase in ASM activity and mRNA expression, ASM knockdown had no effect on RGDfV-induced c-Abl phosphorylation. These data indicate that ASM mediates RGDfV-induced apoptosis and that c-Abl acts upstream of ASM in this apoptotic pathway. This is the first report linking the pro-apoptotic role of c-Abl to ASM and the first to identify c-Abl as a regulator of ASM.

## Materials and Methods

### Apoptosis Assay

Apoptosis was evaluated by flow cytometry in cells (combined adherent and non-adherent) using the Apo-Direct kit (BD Bioscience) detecting terminal deoxynucleotidyltransferase (TdT) activity as incorporation of FITC-dUTP (fluorescein isothiocyanate-deoxyuridine triphosphate) compared with propidium iodide (PI) incorporation according to the manufacturer’s instructions. Apoptosis in cells grown in three-dimensional (3D) collagen type I was evaluated using the AnnexinV-FITC fluorescence microscopy kit (BD Biosciences) according to manufacturer’s instructions as described [33], [34]. Fluorescent images were acquired on an Olympus CKX41 microscope and photographed using PictureFrame 2.1 software (Optronics; original magnification x400) [33].

### ASM Activity Assay

ASM activity was determined using the ASM Assay Kit (#K-3200, Echelon Biosciences, Salt Lake City, UT) according to manufacturer’s instructions, using conditions that yielded readings in the linear range of the assay. Cell lysates (prepared by three freeze/thaw cycles in presence of 1 mM PMSF; 1.2 µg protein/sample) were incubated overnight at 37°C with 50 µl substrate. Fluorescence intensity was read at 360 nm excitation/460 nm emission using a Synergy HT microplate reader (Bio-TEK Instruments, Inc., Winooski, VT). ASM activity was calculated using the standard curve of ASM enzyme activity supplied with the kit and was normalized to protein content (nmol/h/mg sample protein).

### Caspase Activation Assay

Caspase activities were measured using the ApoTarget Caspase-3/CPP32 and Caspase-8/FLICE Colorimetric Protease Assays (BioSource, CA) and were determined in 200 µg lysate protein suspended in 50 µl extraction buffer according to manufacturer’s instructions and as described [33]. Absorbance at 400/405 nm was determined after 16 hrs incubation (37°C) with substrate.

### Cell Culture

The ECV-304 bladder carcinoma cell line (ATCC CRL-1998, Manassas, VA; expresses integrin αvβ3 [35], [36] and data not shown) was maintained in RPMI-1640 with 10% (v/v) fetal bovine serum, 2 mM L-glutamine, 1 mM sodium pyruvate and 20 mM HEPES. For experiments with RGDfV, cells were seeded in serum-free medium on vitronectin-coated plates (2 µg/ml) that were blocked with 1% heat-denatured fatty-acid-free bovine serum albumin (HD-BSA) as described [11], [33], [37]. RGDfV (5 µg/ml) or vehicle were added when cells were attached and spread (2–16 hrs after cells were transferred to Petri dishes). Cells were incubated with RGDfV or vehicle for 24 hrs for western blotting and caspase assays and 48 hrs for flow cytometry assays, or as indicated.

### LC/MS/MS Analysis of Intracellular Sphingolipids

Sphingolipids were determined in duplicate by ESI/MS/MS performed on a ABI 4000Qtrap triple quadrupole mass spectrometer, operating in a multiple reaction monitoring positive ionization mode as described, with moderate modifications [38]. Briefly, 5×10^6^ cells/sample were trypsinized, washed with ice-cold 50 mM Tris-HCl buffer (pH 7.4) containing 150 mM NaCl, 5 mM EDTA, and 5 mM EGTA, and snap-frozen until use. Internal sphingolipid standards consisting of 50 µl mixture containing 1 pM C17-Sphingosine (Sph), C17-dehydrosphingosine (dhSph), C17-sphingosine-1-phosphate (S1P), and C17-Ceramide (Avanti Polar Lipids, Inc, Alabaster, AL) were added to cell pellets before lipid extraction with 4 ml ethyl acetate/isopropyl alcohol/water (60∶30:10%; v/v) solvent system. After centrifugation the organic layer was transferred to glass tubes and evaporated under a stream of air at 40°C. Samples and standards were reconstituted with 4 ml MeOH, 1 ml of the lipid extract was used for determination of phospholipid (Pi) and sphingomyelins as described (30), and the remaining extract was used for quantitation of ceramides by ESI/MS/MS. For ESI/MS/MS, the dried lipid extract was dissolved in HPLC mobile phase (0.5 ml, 1 mM ammonium formate in methanol containing 0.1% v:v formic acid). Lipid samples (10 µl) were injected onto the Agilent 1200 HPLC combined with the ABI 4000Qtrap MS/MS system and gradient-eluted from a BDS Hypersil C8, 150×3.2 mm, 3-µm particle size column, with 1 mM methanolic ammonium formate with 0.1% (v:v) formic acid, 2 mM aqueous ammonium formate with 0.1% formic acid (v:v) mobile phase system. Peaks corresponding to the target sphingolipid analytes and internal standards were collected and processed using the Analyst software system. Quantitative analysis was based on calibration curves generated by spiking an artificial matrix with known amounts of target analyte synthetic standards and an equal amount of internal sphingolipid standards. Target analyte/internal sphingolipid standards peak area ratios were plotted against analyte concentrations, which were normalized to their respective internal sphingolipid standards and compared with the calibration curves, using a linear regression model. Levels of sphingolipids in different cell samples were normalized to Pi.

### Mitochondrial Membrane Depolarization **(** ΔΨm**)**

Mitochondrial membrane depolarization was measured using JC-1 (5,5′,6,6′-tetrachloro-1,1′,3,3′-tetraethylbenzimidazolyl-arbpcyanine iodide; Sigma, St. Louis, MO) as described [33]. Briefly, after incubation with RGDfV cells were resuspended in 0.5 ml serum-free RPMI-1640 containing 10 µg/ml JC-1 and assessed by flow cytometry. Mitochondrial depolarization is represented by cells that shift from red to green florescence. Bandpass filters were 525±25 nm for JC-1 green emission and 610±10 nm for JC-1 red emission.

### Reagents

Ceramides, dihydroceramides, sphingomyelins, sphingoid bases, and their phosphates were from Avanti Polar Lipids, Inc (Alabaster, AL). RGDfV (NSC#707544; Cilengitide) was from the Drug Synthesis and Chemistry Branch, Developmental Therapeutics Program, Division of Cancer Treatment and Diagnosis, National Cancer Institute. STI-571 (imatinib mesylate) was a kind gift from Novartis. Vitronectin was generated as described [11], [33], [37]. Collagen type I used for 3D culture was from BD Biosciences. All other reagents were from Sigma-Aldrich (St. Louis, MO) unless stated otherwise.

### RNA Extraction and Reverse Transcription-Polymerase Chain Reaction **(**RT-PCR**)**

Total RNA was isolated using TRIzol (Invitrogen, Carlsbad, CA). Real time reverse transcription-polymerase chain reaction (qRT-PCR) and Applied Biosystems 7900HT sequence detection system (ABI, Foster City, CA) were used to quantify expression of ASM in ECV-304. Real time qRT-PCR primers and probes for ASM and glyceraldehyde-3-phosphate dehydrogenase (GAPDH) were designed and synthesized by Primer Express software (ABI, Foster, CA) and checked for specificity against GeneBank. Probes and primers used were: ASM, 5′- CCCAATCTGCAAAGGTCTATTCA-3′ (forward primer), 5′- CCCACGC GAGCCACAT-3′ (reverse primer), and 5′- CAACCTCGGGCTGAAGAAGGAA CCC-3′ (probe); GAPDH, 5′- CAACTACATGGTTTACATGTTCCAATATG-3′ (forward primer), 5′-GGGATCTCGCTCCTGGAAG-3′ (reverse primer), and 5′-CGTTCTCAGCCTTGACGGTGCCA-3′ (probe). TaqMan real-time qRT-PCR data were analyzed with the use of ABI Sequence Detector Software. Messenger RNA (mRNA) level for ASM was normalized to mRNA expression of GAPDH, which was quantified in parallel from each sample. For standard RT-PCR, 2.5 µg of RNA was reverse transcribed using oligo(dT)12–18 primer and SuperScript™ III reverse transcriptase (Invitrogen, Carlsbad, CA). One microliter of the reverse transcription product was subjected to PCR amplification. Primer sequences were 5′-CTGACTCTCGGGTTCTCTGG-3′ (ASM forward primer), 5′-AGGTTGAT GGCGGTGAATAG-3′ (ASM reverse primer), 5′-GCCAAAAGGGTCATCATCTC-3′ (GAPDH forward primer), and 5′-GTAGAGGCAGGGATGATGTTC-3′ (GAPDH reverse primer) and produced 160 bp (ASM) and 287 bp (GAPDH) products respectively. For both reactions denaturation was at 95°C for 45 sec, annealing at 56°C for 45 sec, and extension at 72°C for 1 min. ASM was amplified for 27 cycles and GAPDH for 23 cycles, and products were separated on a 1.5% agarose gel.

### RNA Interference

Two specific siRNAs for ASM, HS_SMPD1_1_HP (ASM1) and HS_SMPD1_2_HP (ASM2) and a scrambled control sequence (AllStars negative control siRNA) were from Qiagen (Valencia, CA). c-Abl siRNA (5′-GAAGGGAGGGUGUACCAUUtt-3′) was from Ambion (Austin, TX). ECV-304 (2×10^5^ cells) seeded in 6-well plates and allowed to grow overnight were transfected with 100 pM siRNA or mock-transfected for 5 hrs using Lipofectamine 2000 (Invitrogen, Carlsbad, CA). For RGDfV experiments transfection was done on vitronectin-coated HD-BSA/blocked plates and 48 hrs later RGDfV was added for additional 24 hrs in serum-free medium. ASM and GAPDH mRNA were measured by quantitative real time qRT-PCR. The ASM siRNAs were specific in that they did not affect ERK, GAPDH or β-actin on western blots (not shown).

### SDS-PAGE and Western Blotting

Cell pellets re-suspended in SDS sample buffer were sonicated, boiled 5 min and resolved on a sodium dodecyl sulfate–polyacrylamide gel electrophoresis (SDS-PAGE) gel (10%) as described [11]. Primary antibodies for western blots were: anti-c-Abl 8E9, mouse monoclonal (1∶1000) from BD Biosciences; anti-c-Abl K-12, rabbit polyclonal (1∶1,000); anti-phospho-c-Abl (Y412), rabbit polyclonal (1∶1000) from Abcam; anti-ERK1 K-23, rabbit polyclonal (1∶1,000); anti-GAPDH, mouse monoclonal (1∶20,000) from Santa Cruz Biotechnology (Santa Cruz, CA); anti-poly(adenosine diphosphate-ribose) polymerase (PARP), rabbit polyclonal (1∶1,000) from Cell Signaling Technology (Danvers, MA); anti-β-actin, mouse monoclonal (1∶20,000) from Sigma (St. Louis, MO). Detection was by ECL (Amersham Biosciences, Piscataway, NJ) and densitometry was analyzed by ImageJ (National Institutes of Health).

### Statistical Analysis

Statistical analyses were performed using GraphPad Prism 5.0c for MacIntosh (GraphPad Software, San Diego, CA). All experiments were repeated at least three times unless indicated otherwise. Data are expressed as mean ± standard error of the mean (SEM). P-values were calculated by unpaired t-test unless indicated otherwise, and are detailed in the graphs, legends, or tables. Significance level was set at p<0.05.

## Results

### Inhibition of αvβ3/αvβ5 Integrins by RGDfV Incudes Apoptosis and Upregulates ASM mRNA and ASM Activity

**Figure 1:**
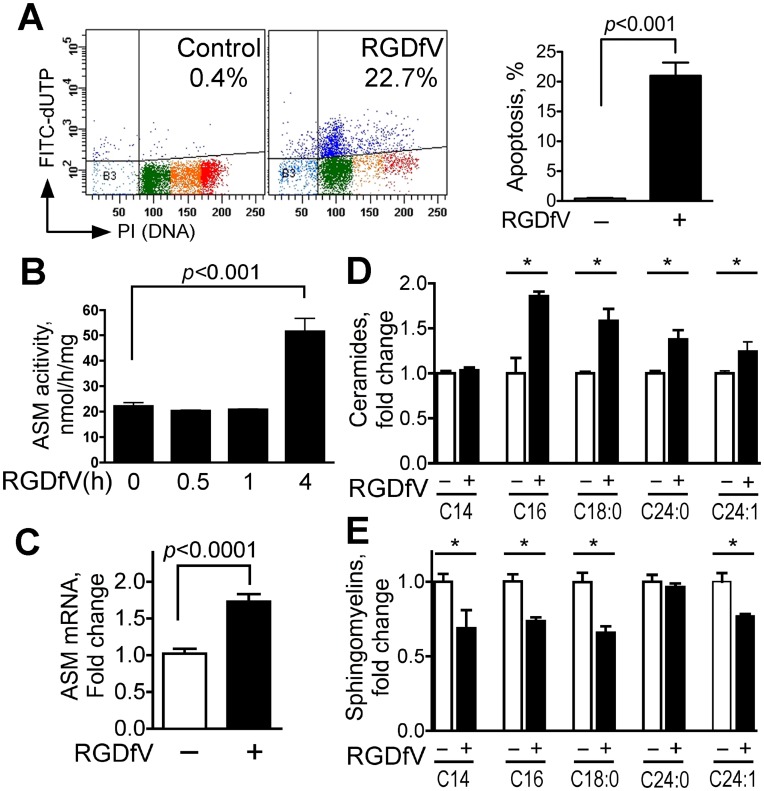
RGDfV increases ASM mRNA, ASM activity and ceramide levels and decreases sphingomyelins. **A**) Apoptosis (Apo-Direct kit) in ECV-304 cells treated with vehicle or RGDfV (5 µg/ml; 48 hrs). Left panel: representative flow cytometry plots showing percentage of apoptotic cells (FITC-dUTP positive cells in the two upper quadrants; dark blue dots). Color dots in the lower quadrants represent position in the cell cycle of the non-apoptotic cells based on DNA content (propidium iodide). Light blue is sub G_0_, green is G_1_, orange is S-phase and red is G_2_/M. Right panel: mean±SEM apoptotic (dark blue) cells of 24 independent experiments. **B**) ASM activity (Echelon Biosciences kit) in extracts from ECV-304 treated with vehicle or RGDfV (5 µg/ml). Shown are mean±SEM of three independent experiments. **C**) Real time quantitative RT-PCR for ASM from ECV-304 cells treated with vehicle or RGDfV (24 hrs). Shown are mean±SEM ASM mRNA normalized to GAPDH, depicted as fold-increase compared to baseline from 16 independent experiments performed in 1–4 replicates each. **D–E**) Change in ceramide (D) and sphingomyelin (E) content in extracts from ECV-304 cells incubated with vehicle or RGDfV (5 µg/ml; 24 hrs) and analyzed by mass spectrometry. Bars represent mean±SEM fold change from control in one of two experiments with similar results, each of which was performed in 3 biological triplicates and analyzed in duplicates.

ECV-304 cells express integrin αvβ3 and αvβ5 [35], [36]. Inhibition of integrins αvβ3 and αvβ5 by RGDfV resulted in apoptosis of ECV-304 cells seeded on vitronectin (Fig. 1A; also supported by PARP cleavage, caspase-3 and caspase-8 activation, mitochondrial depolarization and AnnexinV staining shown in the non-silencing siRNA controls of the third and fourth figures). The response of ECV-304 to RGDfV was associated with a mean 2-fold increase in ASM activity that was detected after four hours or longer of exposure to RGDfV (baseline: 22.1±1.4 vs. at 4h: 51.5±5.3 ng/h/mg protein, p<0.001), but not in the first 5–60 min (Fig. 1B; also supported by the first two columns in panel B of the fifth figure). In view of this relatively late time course, we asked whether the increased activity could be due to change in ASM expression. Indeed, using real time quantitative RT-PCR (qRT-PCR) we consistently observed increase in ASM mRNA in cells incubated with RGDfV (mean 1.70±0.11 fold increase after 24 hrs incubation in 16 separate experiments, each performed in 1–4 replicates; p<0.001), which persisted at least up to 96 hrs incubation (Fig. 1C and data not shown). Consistent with increase in ASM activity and ASM mRNA, mass spectrometry showed that exposure of ECV-304 cells to RGDfV significantly increased level of four ceramide species (C_16_, C_18∶0_, C_24∶0_ and C_24∶1_-ceramide) and decreased sphingomyelins (SM) C_14_-SM, C_16_-SM, C_18∶0_-SM, and C_24∶1_-SM by 18% –35% (Fig. 1D–E and Table 1 NC vs. NC+RGDfV). Thus, inhibition of integrins αvβ3/αvβ5 by RGDfV, that leads to ECV-304 apoptosis, is associated with increased ASM mRNA, increased ASM activity, enhanced hydrolysis of specific sphingomyelins, and increase in ceramides.

**Table 1:** RGDfV increases ceramides and decreases sphingomyelins, and these changes are abrogated following ASM. knockdown.

Ceramides (pmol/nmol Pi)										
	C14-CER		C16-CER	C18:0-CER	C18:1-CER	C20:0-CER	C22:0-CER	C24:0-CER	C24:1-CER	
**NC**	0.44±0.08		**3.17**±**0.13**	**0.10**±**0.01**	0.04±0.01	0.15±0.03	1.85±0.21	**3.35**±**0.35**	**7.83**±**0.59**	
**NC+RGDfV**	0.47±0.07		**4.76**±**0.29**	**0.14**±**0.01**	0.05±0.01	0.13±0.01	1.94±0.14	**4.26**±**0.17**	**10.14**±**1.09**	
**p value**	NS		**0.008**	**0.042**	NS	NS	NS	**0.039**	**0.016**	
									
**ASM1**	0.43±0.05		3.09±0.51	0.1±0.01	0.06±0.01	0.15±0.02	1.85±0.19	2.48±0.17	6.93±0.57	
**ASM1+RGDfV**	0.36±0.02		3.30±0.29	0.1±0.01	0.06±0.01	0.11±0.01	1.94±0.23	3.4±0.16	7.59±0.24	
**p value**	NS		NS	NS	NS	NS	NS	**0.021**	NS	
									
**ASM2**	0.36±0.01		2.60±0.21	0.09±0.01	0.06±0.01	0.14±0.01	1.77±0.14	3.16±0.37	7.07±0.31	
**ASM2+RGDfV**	0.40±0.03		4.13±0.55	0.11±0.01	0.07±0.02	0.12±0.03	1.92±0.29	3.59±0.50	8.61±1.06	
**p value**	NS		0.061	NS	NS	NS	NS	NS	NS	
**Sphingomyelins (pmol/nmol Pi)**										
		**C14-SM**	**C16-SM**	**C18:0-SM**	**C18:1-SM**	**C20:0-SM**	**C22:0-SM**	**C22:1-SM**	**C24:0-SM**	**C24:1-SM**
**NC**		**1.36**±**0.00**	**46.02**±**2.79**	**1.70**±**0.14**	43.83±1.57	4.12±0.39	21.51±1.60	0.6±0.06	**65.11**±**2.62**	**11.47**±**0.69**
**NC+RGDfV**		**0.94**±**0.02**	**38.04**±**0.46**	**1.36**±**0.09**	37.71±1.16	3.07±0.24	17.19±0.59	0.41±0.05	**51.27**±**1.35**	**8.61**±**0.42**
**p value**		**0.001**	**0.047**	**0.035**	NS	NS	NS	NS	**0.018**	**0.024**
									
**ASM1**		1.31±0.08	50.47±1.17	2.28±0.22	59.5±3.06	5.04±0.53	27.67±1.03	0.63±0.10	73.40±2.45	12.29±0.87
**ASM1+RGDfV**		1.23±0.20	51.04±2.61	2.25±0.20	63.94±6.96	4.81±0.34	27.02±2.17	0.57±0.08	73.59±3.99	11.55±0.99
**p value**		NS	NS	NS	NS	NS	NS	NS	NS	NS
									
**ASM2**		1.55±0.06	57.51±10.26	3.63±0.55	89.99±9.11	7.58±1.28	35.79±6.49	0.98±0.19	82.08±15.31	15.09±2.76
**ASM2+RGDfV**		1.23±0.12	56.39±14.60	3.73±1.07	99.79±27.60	7.46±1.88	34.78±8.66	0.90±0.29	79.38±13.78	14.67±4.20
**p value**		NS	NS	NS	NS	NS	NS	NS	NS	NS

**Figure 2:**
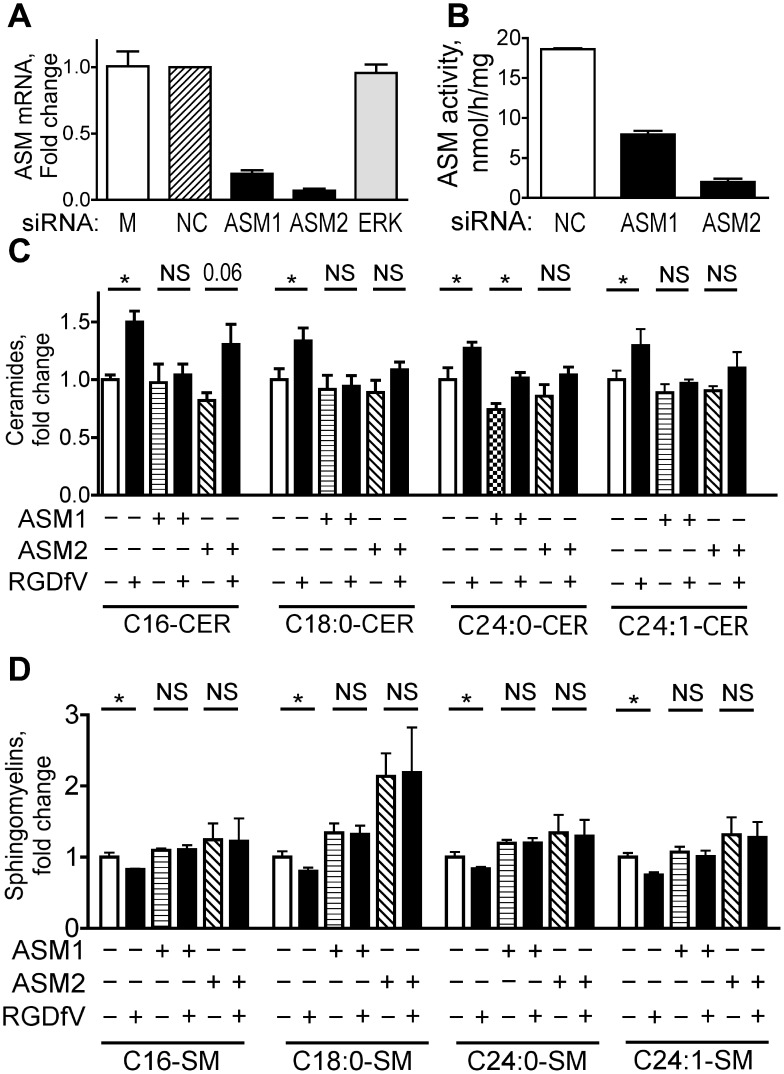
ASM knockdown attenuates the RGDfV-induced changes in ceramides and sphingomyelins. ECV-304 cells were transfected with siRNA as described in Methods. **A**) Messenger RNA extracted from HBMEC 48 hrs after transfection with ASM1, ASM2, ERK, or control siRNA was analyzed for ASM expression by qRT-PCR (n = 6; p<0.001 for ASM1, p<0.001 for ASM2 and p = 0.54 for ERK by unpaired t-test for each siRNA compared to non-silencing control). **B**) ASM activity in extracts of ECV-304 72 hrs after ASM-siRNA transfection compared to NC controls (Echelon Biosciences kit). Shown are mean±SEM; n = 3; p<0.001 for ASM1 and for ASM2, by unpaired t-test. **C–D**) Change in ceramide (panel C) and sphingomyelin (panel D) in extracts of ECV-304 cells after NC, ASM siRNA #1 or ASM siRNA #2, that were then incubated with vehicle or RGDfV (5 µg/ml; 24 hrs) and analyzed by mass spectrometry. Bars represent mean±SEM fold change from control in one of two experiments with similar results, each of which was performed in total of 3 biological triplicates. Absolute lipid values are provided in Table 1.

Lysates of cells treated with siRNA and RGDfV as in Figs. 2C–D as were analyzed by mass spectrometry as detailed in Methods. Sphingolipid nomenclature: Ceramides are 1,3-hydroxy-2-amino alkanes (sphinganine) or alkenes (sphingosine) with fatty acid alkyl chains of C14 to C24 linked to the amino group of the sphingosine or sphinganine. A double bond in fatty acid alkyl chains is indicated as number “1” in the name. For example, C14-CER is sphingosine + C14 fatty acid without a double bond, and C18:1-CER is sphingosine + C18 fatty acid with a double bond. Sphingomyelins (SM) have sphingosine with the 1-hydroxyl group modified to phosphocholine as a core structural moiety, and the numbers for SM are the number of carbons of fatty acid chain. The levels of C20:1-CER, C22:1-CER and C20:1-SM were below the level of detection and were thus were not included in the table. Shown are means of three independent experiments ± SEM. NS: not statistically significant.

### ASM Knockdown Attenuates RGDfV-induced Apoptosis

Acid sphingomyelinase mediates apoptosis in response to a number of stimuli [24], [25], [26], but it has not been definitively shown yet if it mediates RGDfV-induced apoptosis. We therefore knocked down ASM by siRNA and examined effect on RGDfV-induced apoptosis compared to non-silencing control siRNA. The two siRNAs to ASM: #1 (ASM1) and #2 (ASM2), deceased ASM mRNA by 80–90% at 48–72 hrs compared to non-silencing control or ERK siRNA (Fig. 2A and data not shown). Functionally, the decrease in ASM mRNA induced by siRNAs ASM1 and ASM2 (Fig. 2A) was paralleled by decrease in the activity of ASM (Fig. 2B). Last, the ceramide increase and sphingomyelin decrease induced by RGDfV were mostly abolished in cells with siRNA directed against ASM, demonstrating that the RGDfV-induced changes in most of these sphingolipids were induced by ASM (Fig. 2C–D and Table 1).

**Figure 3:**
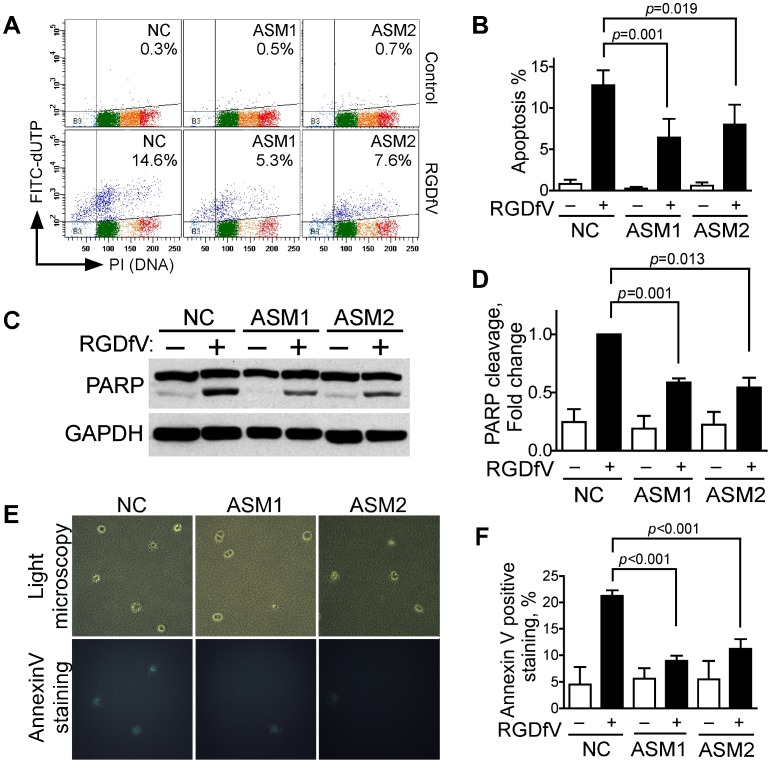
ASM knockdown inhibits RGDfV-induced apoptosis in 2D and 3D conditions. **A–B**) Apoptosis was assessed by flow cytometry (Apo-Direct kit) in siRNA-treated (48 hrs) cells incubated with RGDfV or vehicle (5µg/ml; additional 48 hrs). **A**) Representative experiment of three with similar results; the percent apoptotic cells indicated is sum of both upper quadrants. Color dots are as detailed in Fig. 1A. **B**) Mean±SEM of three experiments as shown in panel A. **C–D**) PARP cleavage (western blotting) in lysates of cells treated with siRNA (48 hrs) and incubated with RGDfV (5µg/ml) or vehicle for additional 24 hrs. Panel D provides densitometry of PARP cleavage showing mean±SEM of three similar experiments. **E–F**) AnnexinV-FITC staining of ECV-304 (5×10^4^ cells/ml) cultured in 3D-collagen, treated with siRNA to ASM or non-silencing control (NC) and incubated 24 hrs with RGDfV (5 µg/ml) or vehicle. **E**) Representative fields (top panels: light microscopy, bottom panels: fluorescence) in presence of RGDfV. Control panels without RGDfV had only rare AnnexinV-positive cells and are not shown. **F**) Effect of ASM siRNA on percent of AnnexinV-positive cells relative to total cells in the same fields. Total cells counted on bright field images per sample were between 300–800 cells. Original magnification: 400x. p<0.001. **NC**: negative control (non-specific non-silencing siRNA); **ASM1**: siRNA#1 to ASM; **ASM2**: siRNA#2 to ASM.

To determine if ASM mediated the RGDfV-induced apoptosis we next tested if ASM siRNA would block RGDfV-induced apoptosis in ECV-304 cells. Downregulation of ASM by siRNAs ASM1 and ASM2 decreased TUNEL stain induced by RGDfV compared to non-specific siRNA negative controls (Fig. 3A–B). Further, siRNA to ASM mitigated the cleavage of PARP induced by RGDfV (Fig. 3C–D). Last, in cells cultured in a 3D-collagen matrix, simulating more physiological conditions compared to two dimensional culture plates, inhibition of integrins by RGDfV in control cells (treated with nonspecific non-silencing siRNA) increased FITC-AnnexinV-positive cells more than four-fold (p<0.001). However, if ASM was knocked down by siRNAs, the RGDfV-induced increase in AnnexinV-positive cells was abolished (p = 0.11 for siRNA ASM1 and p = 0.13 for siRNA ASM2 using vehicle vs. RGDfV-treated cells; Fig. 3E–F). Complementing this, the increase in AnnexinV-positive cells induced by RGDfV in ASM-siRNA cells was blocked compared to control siRNA cells (p<0.001; Fig. 3F).

**Figure 4:**
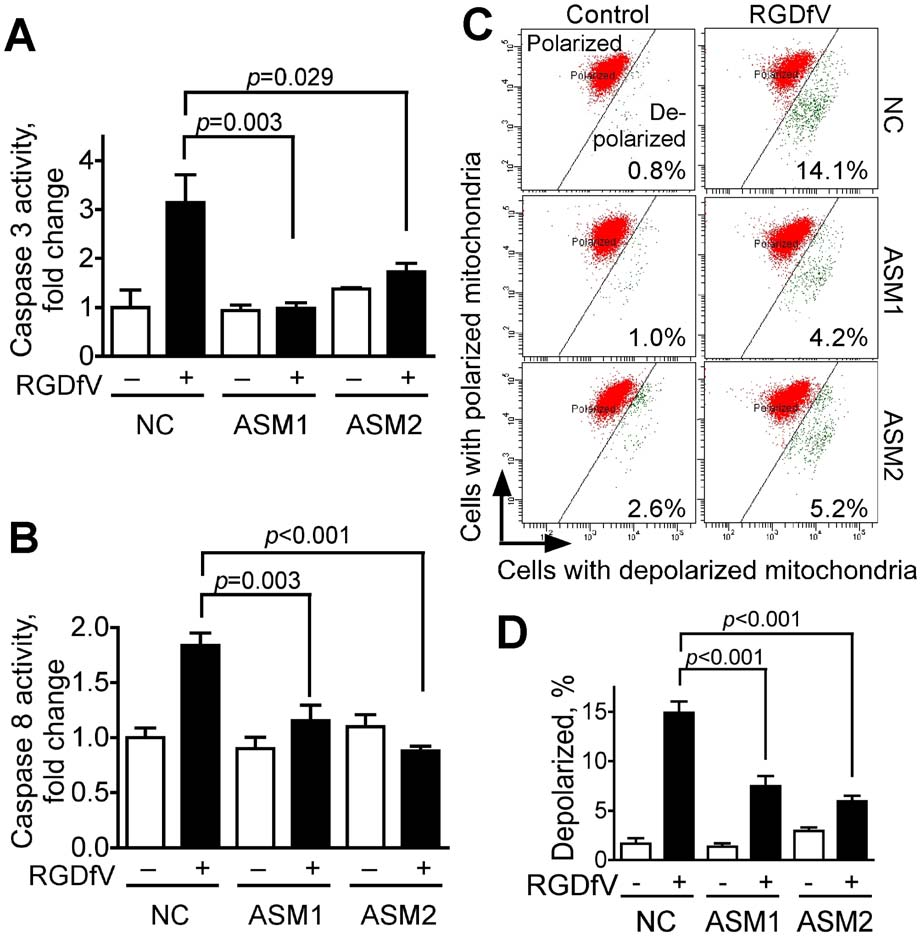
ASM knockdown inhibits RGDfV-induced activation of caspase-3 and caspase-8 and diminishes RGDfV-induced disruption of mitochondrial membrane potential. ECV-304 cells transfected 48 hrs with non-specific non-silencing negative control siRNA, ASM1-siRNA or ASM2-siRNA were treated with vehicle or RGDfV (5 µg/ml; 24 hrs). **A–B**) Lysates analyzed using the ApoTarget caspase-3/CPP32 (A) or caspase-8/FLICE (B) colorimetric protease assays. Bars represent mean±SEM; n = 6 for each condition. **C–D**) Loss of mitochondrial membrane potential (ΔΨm) was measured by flow cytometry using the JC-1 mitochondrial probe. Panel C shows representative flow cytometry plots. Red: mitochondria with normal polarization, green: depolarized mitochondria. Values in the panels represent percentage of cells with depolarized mitochondrial membrane. Panel D shows percent of cells with depolarized mitochondrial membrane potential from 3 independent experiments, each performed in triplicate (means ± SEM).

We next tested if ASM-siRNA could alter RGDfV-induced caspase cleavage, caspase activity and mitochondrial membrane depolarization in ECV-304 compared with non-silencing control siRNA (Fig. 4). RGDfV increased activity of caspase 3 and caspase 8 in non-silencing siRNA cells to 310±35% and 180±30% of control, respectively (Fig. 4A–B). However, in siRNAs ASM1- and ASM2-treated samples, the RGDfV-induced activation of both caspase 3 and caspase 8 (Fig. 4A–B) was significantly diminished, demonstrating that ASM functions upstream of caspase 3 and caspase 8 and is required for their activation during RGDfV-induced apoptosis.

RGDfV-induced apoptosis was also associated with mitochondrial depolarization as measured by the JC-1 mitochondrial probe (Fig. 4C–D). Downregulation of ASM using ASM1 or ASM2 siRNAs, but not non-specific non-silencing siRNA, diminished this RGDfV-induced mitochondrial membrane depolarization by up to 50–60% (Fig. 4C–D). These results place ASM upstream of mitochondrial membrane depolarization in RGDfV-induced apoptosis.

Taken together, these data demonstrate that increase in ASM mediates the apoptosis induced by RGDfV, and that ASM functions upstream of caspase 8, caspase 3 and mitochondrial depolarization.

### Inhibition of c-Abl Attenuates RGDfV-induced Increase in ASM mRNA and ASM Activity

In Figs. 1, 2, 3, 4 we showed that increased ASM is required for apoptosis induced by RGDfV in ECV-304 cells. We recently showed that RGDfV induces phosphorylation of c-Abl on tyrosines Y412 and Y245 and that c-Abl is required for RGDfV-induced apoptosis [33]. This raised the question of whether c-Abl and ASM signaling were functionally-related in RGDfV-induced apoptosis or were independent of each other.

**Figure 5:**
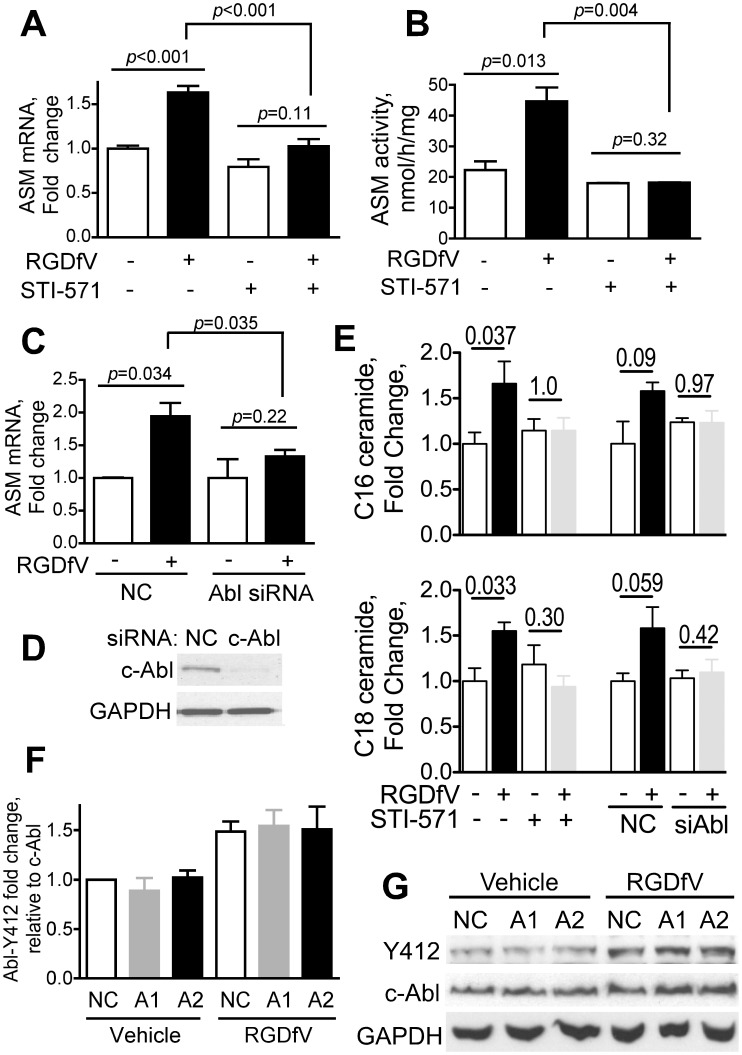
Inhibition of c-Abl abrogates the RGDfV-induced increase in ASM mRNA and ASM activity. **A–B**) ECV-304 cells were treated with STI-571 (c-Abl inhibitor; 10 µM) or vehicle starting 2 hrs before adding RGDfV (5 µg/ml) or vehicle for additional 24 hrs. (**A**) ASM mRNA (real time qRT-PCR; mean±SEM of 4 independent experiments performed in 2 replicates), (**B**) ASM activity (Echelon Biosciences kit; mean±SEM of 2 independent experiments performed in 4 replicates). **C–D**) ASM mRNA (real time qRT-PCR relative to GAPDH) of ECV-304 transfected with non-specific non-silencing negative control siRNA (NC) or c-Abl siRNA (48 hrs) and incubated with RGDfV (5 µg/ml) or vehicle for 24 hrs. Shown are mean±SEM, n = 4. Representative efficacy of c-Abl siRNA knockdown (western blot) is shown in Panel D. **E**) Change in ceramide content in extracts from cells incubated with STI-571 or siRNA-c-Abl and their controls with/without RGDfV as in panels A–D was analyzed by mass spectrometry. Bars represent mean±SEM fold change in ceramides C_16_ and C_18_ from four (STI-571) and three (siRNA, either non-silencing controls NC, or c-Abl) independent experiments. Changes in other ceramides were not significant. P-values were calculated using paired t-tests. **F–G**) Phospho-c-Abl (Y412) and total c-Abl (western blotting) in lysates of ECV-304 transfected with NC, ASM1 or ASM2 siRNA as in Fig. 3 and treated 24 hrs with RGDfV (5 µg/ml) or vehicle. GAPDH served as loading control. Panel **G** shows a typical experiment and panel **F** shows mean±SEM of phospho-c-Abl (Y412) relative to total c-Abl from densitometry of three experiments. ASM mRNA level in these experiments was 28±1.2% of the non-silencing control samples for siRNA-ASM1 and 11.4±0.9% for siRNA-ASM2.

We first tested the effects of the c-Abl inhibitor STI-571 (imatinib) on ASM (Fig. 5A–B). We found that STI-571 inhibited both RGDfV-induced increase in ASM mRNA expression (Fig. 5A, p<0.001) and the associated increase in ASM activity (Fig. 5B, p = 0.004), suggesting that ASM is regulated by c-Abl. c-Abl siRNA also mitigated the RGDfV-induced increase in ASM mRNA (Fig. 5C), further supporting that c-Abl participates in regulation of ASM. Efficacy of c-Abl siRNA knockdown is shown in Fig. 5D). Consistent with these data, inhibition of c-Abl by STI-571 abrogated the increase in ceramides C_16_ and C_18_ that were observed in presence of RGDfV in cells with unhindered c-Abl (Fig. 5E). Similar experiments using siRNA showed that whereas RGDfV tended to increase ceramides C_16_ and C_18_ in cells with non-silencing control siRNA (p-values approaching significance), in cells with knocked down c-Abl there was no difference in ceramides C_16_ and C_18_ in presence or absence of RGDfV (Fig. 5E). siRNAs to ASM (ASM1, ASM2) however, were not able to block RGDfV-induced phosphorylation of c-Abl at Y412, nor did they change c-Abl protein expression (Fig. 5F–G). This molecular ordering of c-Abl and ASM places c-Abl as an upstream regulator of RGDfV-induced increase in ASM expression and ASM activity.

**Figure 6:**
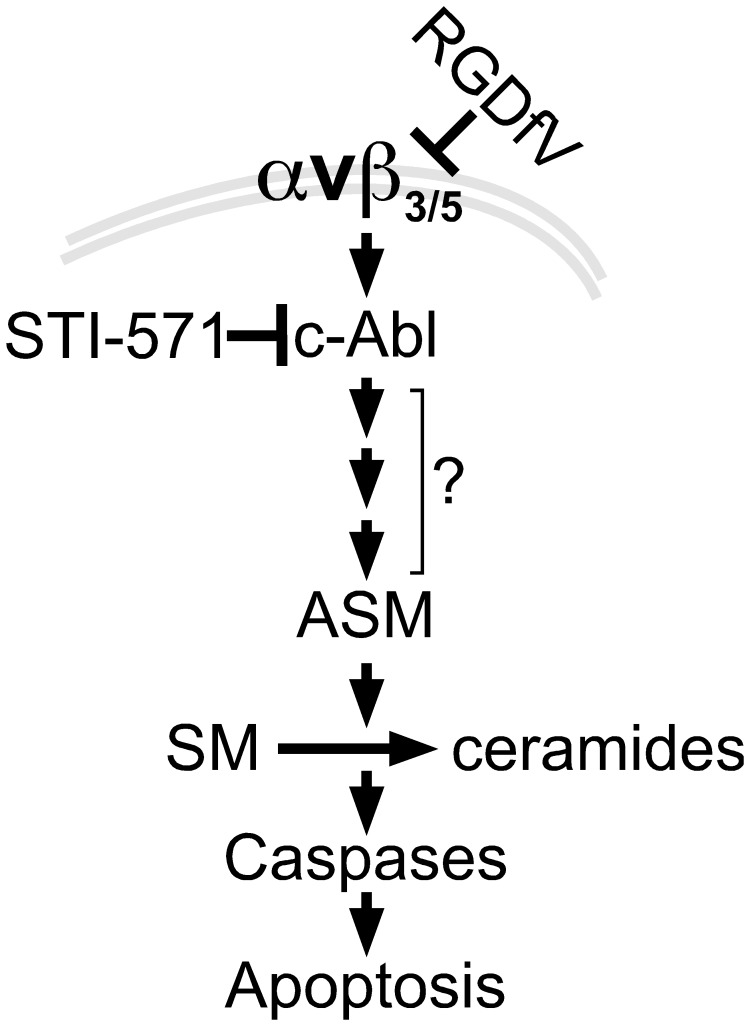
Model to link RGDfV-induced integrin αvβ3/αvβ5 inhibition, c-Abl, ASM and apoptosis. Our data support a model in which inhibition of integrin αvβ3/αvβ5 signaling by RGDfV activates c-Abl (26). c-Abl in turn increases expression of ASM, which results in increased ASM activity and changes in sphingolipids, including increase in ceramides and decrease in sphingomyelins. The consequence of the increase in ASM expression and activity is apoptosis. Since ASM mediates the apoptosis, but it is not known which sphingolipid(s) is/are the mediator(s) of the apoptosis downstream of ASM, the downstream arrows are placed below ASM rather than below specific sphingolipids. Since downregulation of ASM expression leads to inhibition of caspase 3 and caspase 8 activity, caspases are placed downstream of ASM. Inhibition of c-Abl blocked the increase in ASM mRNA and activity and prevented the apoptosis, placing c-Abl upstream of ASM. It is likely that the interaction between c-Abl and ASM is not direct and that other yet-unknown mediators (denoted by the three small arrows and question mark) exist within this pathway. Not shown are possible initial upstream regulation of ASM by caspase 8 and other intermediate effectors.

In summary, our results in ECV-304 demonstrate that inhibition of integrins αvβ3/αvβ5 by RGDfV increased ASM mRNA and ASM activity and was associated with decreased sphingomyelin and increased ceramide. This increase in ASM was required for the RGDfV-induced apoptosis. Importantly, the increased expression and activity of ASM were mediated by c-Abl, establishing c-Abl as an upstream regulator of ASM in this apoptosis (Fig. 6).

## Discussion

Our study demonstrated a causal role for ASM in apoptosis induced by integrin αvβ3/αvβ5 inhibition by RGDfV in ECV-304 carcinoma cells and showed that the increase in ASM was regulated by c-Abl.

### RGDfV Increases Acid Sphingomyelinase Expression

Our findings add integrin αvβ3/αvβ5 inhibition to other stimuli in which ASM mediates apoptosis [39] and for the first time provide quantitation of changes in ceramide and sphingomyelin following exposure to RGDfV. These findings are consistent with those of Chudakova *et al*, who showed that ASM mediates apoptosis induced by knockdown of αv-integrins in oligodendrocytes [10]. Of note, pharmacological inhibition of integrins αvβ/αvβ5 (i.e., RGDfV), which *in vivo* inhibits angiogenesis and tumor growth, may be biologically different from genetic knockdown of αvβ3/αvβ5, which can result in enhanced angiogenesis [40]. Thus, it is interesting that both are mediated by ASM.

Our finding, that the increase in ASM activity was only detected after hours of RGDfV treatment (Figs. 1B, 2C, 5B) suggests that the increased ASM activity may be at least in part due to the increase in ASM mRNA. This time frame is consistent with results reported by Chudakova *et al*, who demonstrated that siRNA-mediated knockdown of integrin αv in oligodendrocytes was associated with late (48 hrs) sphingomyelin hydrolysis and ceramide accumulation [10]. A second possible explanation may be that the gradual effect of the stimulus (RGDfV, or siRNA to αv-integrin [10]) may contribute to this relatively late timeline, although the effect of RGDfV on cell morphology and integrin inhibition occurred within several hours.

Our results show a 50% increase in ASM mRNA following RGDfV (Fig. 1C), but >2-fold increase in ASM activity (Fig. 1B). This may suggest that in addition to the increased ASM mRNA, there may be further regulation of ASM activity by post-translational modification(s), such as the PMA-induced PKCδ-mediated serine 508 phosphorylation of ASM described by Zeidan and Hannun [41].

Our result place caspase 8 downstream of ASM. This is reminiscent of the mechanism of CD95-induced apoptosis, where initially caspase 8 is mildly activated (∼1% of maximal activity), causing activation and translocation of ASM, and then achieves maximal activation following ASM-induced ceramide generation [42].

### Molecular Ordering of c-Abl and ASM in RGDfV-induced Apoptosis

Our data place c-Abl upstream of ASM in RGDfV-induced ECV-304 apoptosis. To date, there have been no other reports implicating both c-Abl and ASM in the same pathway in apoptosis. The only other studies linking sphingolipid metabolism to Abl were performed in the context of the oncogenic forms of Abl (BCR-ABL1 and v-Abl), where Abl was protective rather than pro-apoptotic. For example, both BCR-ABL1 and v-Abl protected leukemia cells from ASM-mediated Fas-induced apoptosis [43]. A possible explanation for the difference may be that oncogenic forms of Abl do not undergo the nuclear translocation required for manifestation of the growth-inhibitory and/or apoptosis-mediating effects of c-Abl [30], [33], [44]. Therefore, our findings are the first to demonstrate a functional link between c-Abl and ASM and to provide their molecular ordering in apoptosis.

A role for c-Abl in regulation of ASM in apoptosis is supported by reports of several investigators using different cell types, which implicate either c-Abl or ASM in cisplatin-induced apoptosis. Zeidan *et al* demonstrated cisplatin-induced transient activation of ASM in MCF-7 cells [45] and Rebillard *et al* showed that ASM-deficient mice were protected from cisplatin-induced gastrointestinal damage [46]. Gonfloni *et al* then showed that cisplatin-induced apoptosis was mediated by c-Abl, by inducing accumulation of p63 in mouse oocytes [47]. It would be interesting to examine if c-Abl also regulates ASM in cisplatin-induced apoptosis or other apoptotic stimuli that involve c-Abl and/or ASM.

As noted, c-Abl mediates cisplatin-induced mouse oocyte apoptosis by causing accumulation of p63, a p53-family member [47]. p53 can mediate the modulation of DNA damage-induced apoptosis by cell adhesion [48]. c-Abl, a modulator of DNA damage-induced apoptosis, can also mediate this adhesion-dependent modulation of apoptosis, but in a p53-independent manner [49]. c-Abl also regulates p73, another p53 family member, in the apoptotic response to cisplatin-induced DNA damage, in which ASM plays a role [45], [50]. In endothelial cells, p53 is activated following inhibition of integrins αvβ3/αvβ5 by RGDfV [51], suggesting possible interactions between p53 and c-Abl, and/or ASM. Examination of change in p53, p63, or p73 protein expression following manipulation of c-Abl (siRNA or STI-571) in RGDfV-induced apoptosis will be interesting, but will require use of cells other than ECV-304, which have truncated p53 due to an in-frame deletion of tyrosine 126 [52].

c-Abl, typically evoked for its proliferative effects [28], [29], [53] and as mediator of DNA damage-induced apoptosis [30], [31], is also an important regulator and target of cytoskeletal dynamics [54], [55], [56]. A number of studies have also linked ASM to cytoskeletal control: Zeidan *et al* elegantly demonstrated that cisplatin-induced cytoskeletal remodeling in MCF-7 breast adenocarcinoma cells was mediated by transient activation of ASM, which in turn caused dephosphorylation (inhibition) of another actin-binding protein, ezrin (at threonine 567) [45]. The functional output in that study was cytoskeletal remodeling and motility, both of which occur at earlier time points than apoptosis [45]. Rebillard *et al* recently showed that cisplatin-induced apoptosis required F-actin cytoskeletal reorganization mediated by Fas, and that ezrin was also required for that apoptosis [57]. Interestingly, integrin αvβ3 binding to vitronectin causes phosphorylation of ezrin [58]. It remains to be seen whether ezrin will also emerge as an effector in the signaling cascade initiated by integrin αvβ3 inhibition and mediated by c-Abl and ASM, which we identified in our current work.

Taken together, we have demonstrated that apoptosis induced by RGDfV is mediated by increase in ASM mRNA and ASM activity through a c-Abl-dependent mechanism (Fig. 6). These data link for the first time the pro-apoptotic function of c-Abl to ASM-dependent apoptosis and establish the molecular ordering of c-Abl and ASM in apoptosis induced by inhibition of integrins αvβ3/αvβ5.
